# Prevalence of premarital sexual practice and associated factors among undergraduate health science students of Madawalabu University, Bale Goba, South East Ethiopia: institution based cross sectional study

**DOI:** 10.11604/pamj.2015.20.209.4525

**Published:** 2015-03-06

**Authors:** Tomas Benti Teferra, Asfew Negaro Erena, Anteneh Kebede

**Affiliations:** 1 Mada Walabu University, College of Medicine and Health Sciences, Department of Nursing, Bale Goba, Ethiopia; 2 Mada Walabu University, College of Medicine and Health Sciences, Department of Medicine, Bale Goba, Ethiopia

**Keywords:** Premarital sexual practice, undergraduate, sexuality, reproductive health

## Abstract

**Introduction:**

Several studies in Sub- Saharan Africa have documented high and increasing premarital sexual activities among adolescents. Younger people face social, peer and cultural pressure to engage in premarital sex. As a result, significant numbers of adolescents are involved in sexual activities at an early age which exposes them to the risk of unintended pregnancy, early marriage, abortion and STIs/HIV/AIDS. This study was conducted to determine Prevalence of premarital sexual practice and associated factors among Health science students of Madawalabu University, Ethiopia.

**Methods:**

A Descriptive cross sectional survey was employed and three hundred twenty four students were randomly selected after proportional allocation according to their level of education. Data were collected by a self –administered questionnaire and analyzed using SPSS Version 16. A stepwise logistic regression with forward method was used to identify independent predictors of premarital sexual practices at 95% CI and P value less than 0.05.

**Results:**

Of respondents 181 (59.9%) who had a boy or girl friends; about 129 (42.7%) have had premarital sexual intercourse. Out of sexually active respondents, 85 (66.4%) had one sexual partner, 44 (33.6%) had two or more sexual partners. The average age of starting sexual intercourses was 18.4 ±2.14years. Sixty three (20.9%) of respondents reported tobacco smoking and 117 (38.7%) reported consumption of alcohol consumption.

**Conclusion:**

Alcohol use, boarding, sex, educational level and discussion about sexuality were significantly associated with premarital sexual intercourses. So, there is the need to step up Reproductive health club at the university to bring behavior change among the students in order to detain the usual consequences of premarital sexual practices and risky sexual behavior

## Introduction

Adolescence is a stage which human beings face once throughout a lifetime. This stage serves as a threshold for biological, physical, psychological and social developments which are accompanied by either positive or negative behaviours depending on the environment that the child is brought-up [1]. Risky sexual behaviours, including early sexual debut, unprotected sexual intercourse, and multiple sexual partners, occur in a broader context. The intensity of involvement in such behavior ranges from nonsexual relationship to unprotected sexual intercourse with multiple partners and prostitution [2]. Sexual activities among adolescents have been reported to be increasing worldwide. Several studies in Sub- Saharan Africa have also documented high and increasing premarital sexual activities among adolescents [3].

Younger people often face with strong social, peer and cultural pressure to engage in premarital sex [3]. As a result of this, significant numbers of adolescents are involved in sexual activities at an early age [4]. The early sexual activity of young people can expose them to the risk of unintended pregnancy, early marriage, abortion and STIs/HIV/AIDS [5]. In most Sub-Saharan African countries, less than one-third of sexually experienced adolescent girls report using a condom during their most recent sexual experience [6]. In addition, unwanted pregnancy among female students may lead to school dropout, illegal and unsafe abortion, even death [7]. Many adolescents face pressures to use alcohol, cigarettes, or other drugs and to initiate sexual relationships at earlier ages, putting themselves at high risk for intentional and unintentional injuries and risky sexual behaviours [8]. Associations between sexual activity and substance use have been a consistent research finding [9] and these practices may influence them to engage in premarital sex and its complications.

Studies have publicized that about 60% of pregnancies are unwanted or unintended in Ethiopia [3]. According to EDHS 2005, 0.4% of those between the age of 15-19 and 1.1% of those between 20 to 24 years were living with HIV/AIDS [10]. Besides, 14% of all unsafe abortions in low and middle income countries are among women 15-19 years [11] whereas 25-57.5% of induced abortion in Ethiopia occurs among young women aged 15-20 years [12]. So assessing magnitudes of premarital sexual practice and its associated factors is important in newly established university like Madawalabu University of Ethiopia which admits a group of young people who came from different areas with different background. The finding also helps the organization to establish students Reproductive health club that will be work on improve knowledge of school adolescents on reproductive health issues.

## Methods

**Study area and period:** The study was conducted in Madawalabu University, which is one of currently established university in Ethiopia. It is found in Oromia region Bale zone 440KM away from the capital Addis Ababa. The study was conducted from March 1 - may30 2013.

**Study design:** Cross sectional study was employed.

**Population, sample size determination and sampling techniques:** The populations for this study were health science students taken from Madawalabu University, Bale robe Ethiopia. Samples of 324 undergraduate were taken randomly after stratification was made according to their academic level.

**Data collection instrument and techniques:** The instrument consists of semi- structured questions developed by the researchers. It has three parts (sociodemographic data, substances uses and questions asking about sexual history of the students). Data were collected by distributing the tool to randomly selected students and Recollected from the after they filled out.

**Study variables:** Independent variables were socio demographic (age, Sex, current marital Status, educational level) source of information on sexuality and RH, Communication with parents & peers about sexuality and RH and risky behaviors like tobacco uses and alcohol consumption. The dependent variable was premarital sexual practices.

**Data quality control measures:** A pre tested and semi-structured questionnaire was used to collect the data. Before distribution of questionnaires to respondents, they were told about the objective of the study and their importance to participate in the study. Students were told only to report their own experience according to the questions. Then questionnaires were checked for completeness while being taken from the respondents and corrections were made accordingly.

**Data processing and analysis:** Data were entered, cleaned and analysis using SPSS Version 16. Uni variate and bi variate analysis was done. Stepwise logistic regression with forward method was used to identify independent predictors of premarital sexual practices at 95% Confidence interval and P value of ≤ 0.05

**Ethical consideration:** The study was reviewed and approved by the research committee of college of medicine and health sciences of Madawalabu University. Moreover, Respondents were assured of information provided and giving the choice not to partake in the study. Informed consent was obtained from each study subjects. In order to keep confidentiality of the data, name was not included.

## Results

**Results:** From a total of 324 students who were included in the study, 302 participated. The overall response rate was 93.2%. Two hundred sixty six (88.1%) of the respondents were in the age group of 15-24 years. About 221(73.2%) respondents were Christians followed by Muslims religion followers 69 (22.8%). From these about 262 (86.8%) of the respondent attend religious service currently. The majority of the students were from urban area of the country which was about 211(69.9%). Eighty seven (28.8%) of the participants had illiterate mother and 39(12.9%) of the respondent′s fathers were illiterate (Table 1).

**Table 1:** Distribution of respondents by socio- demographic characteristics 2013

Variables	Category	Frequency	%
Age	15-24	266	88.1
	25-34	36	11.9
Sex	Male	222	73.5
	Female	80	26.5
Boarding	Dormitory	290	96.0
	Non dormitory	12	4.0
Religion	Christian	221	73.2
	Muslim	69	22.8
	Others **	12	4.0
Current marital status	single	272	90.1
	Married and living with	13	4.3
	Married and not living with	17	5.6
Batch/academic level	First year	40	13.2
	Second year	90	29.8
	Third year	73	24.2
	Fourth year	99	32.8
Place of origin	Urban	211	69.9
	Rural	91	30.1
Attend religious services currently	Yes	262	86.8
	No	40	13.2
Mother educational status	illiterate	87	28.8
	read and write	70	23.2
	Primary(1 to 8 grade)	52	17.2
	Secondary(9 -12)	23	7.6
	Tertiary(college and above)	70	23.2
Father educational status	illiterate	39	12.9
	read and write	82	27.2
	primary(1 to 8 grade)	48	15.9
	secondary(9 -12)	35	11.6
	tertiary (college and above)	98	32.5

** Others includes waqefeta& catholic

**Risk behaviours of study subjects:** This study revealed that 63 (20.9%) of respondents reported cigarette consumption either daily 12(19.04%), 5-6 days per week 10(15.87%), 1-4 days per week 7(11.11%), 1-3days per week 18(28.57%) or less than once per month 16 (25.39%) and About 117 (38.7%) reported consumption of alcohol (Table 2).

**Table 2:** Distribution of respondents according to alcohol, tobacco and chat uses March, 2013

Variables	Frequency	percentage
Smoke any tobacco products		
Yes	63	20.9
No	239	79.1
How frequently		
Daily	12	19.04
5-6 days week	10	15.87
1-4 days	7	11.11
1-3days per week	18	28.57
less than once per month	16	25.39
Drink any alcohol products		
Yes	117	38.7
No	185	61.3
How frequently		
Daily	5	4.3
5-6 days week	4	3.4
1-4 days	3	2.6
1-3days per week	32	27.4
less than once per month	73	62.4

**Sexual behavior of study subjects:** This study found from the total respondents 181 (59.9%) who had boy or girl friends; about 129 (42.7%) have had premarital sexual intercourse. The average age of starting sexual intercourses for male was 18.4 ±2.14 and 18.2 ±1.62 for female students. From the total sexually active respondents, 85 (66.4%) had one sexual partner, 44 (33.6%) had two or more sexual partners. Concerning Sources of information about sexuality and Reproductive Health- about 288(95.4%) had the information while 14(4.6%) did not. The main sources mentioned by students were health professionals, family and friends, Mass media, school and Religious leaders (Table 3). They were also asked whether used condom during the sexual intercourses and about 70 (54.7%) were did not used. The main reasons given for not using condom includes trust partner 21 (27.3%), partner refused 14 (18.2%), use of other contraceptive13 (16.9%), decrease satsification18 (23.4%) and drunk 4 (5.2%). About 35(31.8%) of sexually active male respondents had history of sexual intercourse with commercial sex workers of which only 2 students were did not used condom during that intercourses (Table 4). Result from multiple logistic regression showed that the odd of having premarital sexual intercourse among students living out of campus, drunken alcohol, stayed two years in the campus and discussing sexuality and reproductive issue were more likely compared to their counterparts (Table 5).

**Table 3:** Premarital sexual histories among Madawalabu University College of medicine and health sciences students, March 2013

Variables	frequency	%
**Have a boy or girl friend**		
yes	181	59.9
no	121	40.1
**Ever had sexual intercourse**		
Yes	129.0	42.7
No	173.0	57.3
**Age at first intercourse**		
15-19	89	69.5
20-24	39	30.5
**Reason for the event**		
Fall in love	84	42.2
Desire for benefit	63	31.7
married	20	10.1
Drunk and stoned	16	8.0
raped	2	1.0
Peer pressure	14	7.0
**Number of partner**		
Only one	85	66.4
Two and above	44	33.6
**Did you use condom during sexual intercourse**		
Yes	70	54.7
No	59	45.3
**Discuss sexual related issues with your family/friends**		
Yes	167	55.3
No	135	44.7
**Sexual intercourse with commercial sex worker (for sexually active male)**		
yes	35	31.81
no	75	68.18
**Use of condom during sexual intercourse with commercial sex worker**		
Yes	33	94.3
no	2	5.7
**Do you have information about reproductive health and sexuality**		
Yes	288	95.4
No	14	4.6

**Table 4:** Main risky behaviors related to pre marital sexual practices among MWU college of medicine health science students, March 2013

Variables	Response category	N	%
Did you use condom during sexual intercourse	Yes	70	54.7
	No	59	45.3
Reason mentioned of not using condom	Ashamed To Ask Partner	1	1.3
	Ashamed To Buy	6	7.8
	Trust Partner	21	27.3
	Partner Objected	14	18.2
	Used Other Contraceptive	13	16.8
	Drunk	4	5.2
	Decrease Satisfaction	18	23.4
	Total	77	100
Sexual intercourse with commercial sex worker	yes	35	31.8
	No	75	68.2
Use of condom during sexual intercourse with commercial sex worker	Yes	33	94.3
	No	2	5.7

**Table 5:** Independent predictors of premarital sexual intercourse among Madawalabu University, college of medicine and health science students, March, 2013

Variables	Unadjusted OR(95%CI)	p-value	adjusted OR(95%CI)	P value
**Boarding**				
dormitory	R		R	
Non dormitory	7.12(1.54-33.38)	0.012	8.64(1.62-46.06)	0.01
**Batch**				
First year	R		R	
Second year	3.93(1.68-9.21)	0.002	5.88(2.04-16.89)	0.00
Third year	2.54(1.05-6.10)	0.037	3.60(1.18-11.00)	0.02
Fourth year	2.43(1.04-5.65)	0.039	3.99(1.40-11.35)	0.01
**Sex**				
Male	R		R	
Female	0.32(017-0.56)	.000	0.24(0.12-0.52)	0.00
**Has boy/girl friend**				
No	R		R	
Yes	4,49(2.67-7.55)	.000	6.45(3.33-12.50)	0.00
**Smoke tobacco/alcohol**				
No	R		R	
Yes	8.77(4.42-17.39)	.000	10.4(4.69-23.11)	0.00
**Discuss sexuality and RH issue with family/relatives**				
No	R		R	
Yes	2.40(1.49-3.86)	0.000	1.84 (1.02-3.32)	0.04

R = reference group, OR = odd ratio, I = confidence interval

## Discussion

Several studies in Sub- Saharan Africa have also documented high and increasing premarital sexual activities among adolescents [2]. This study found that from the total respondents who had boy or girl friends; about 129 (42.7%) have had premarital sexual intercourse. This shows that more young people in higher institutions are getting sexually active and often take advantage of freedom from direct parental supervision and guidance to express their freedom by initiating sexual activity without adequate protection [13]. Indeed, higher institutions give high level of personal freedom and social interactions, which offers an opportunity for high level of sexual networking [14]. On the other hand, young people often face enormous peer's pressure to engage in sex, to watch unlicensed erotic/romantic video films and the desire for some benefit gain. As a result of this, a significant number of adolescents are involved in sexual activities at an early age [15]. The finding from this study is lower than finding of John Imaledo et al (52%) [16], Fawole AO, Ogunkan DV and Adegoke GS. (72.2%) [17]. On the other hand, this finding is relatively higher than that HAPCO in oromia region (31.3%) [18] and result among School Adolescents in Nekemte Town, East Wollega which revealed that about (21.5%) of the participants had had premarital sexual intercourse at the time of the survey [19].

Overall the proportion of sexually active male respondents was higher 110 (49.5%) than that of females 19 (23.8%) which is higher than finding of Dawud A. (9.6% for boys and & 7.7% for females) [20]. In Addis Ababa the proportion premarital sexual practice was 39.8% for males and 5.6% for females [21] and In Gondar, it was 46.2% and 16.2% for males and females, respectively [20]. This may be due to difference in life style of students from different background and accessibility to different factors which put them at risk of having sexual intercourses. Study conducted among students of tertiary institutions in Rivers state found that about 57.0% of students reported having sex without condom and 42.1% reported having had multiple sexual partners [22] while this study found that about 45.3% students had sexual intercourse without a condom and 33.6% has more than one sexual partner. This indicates that lack of adequate knowledge about reproductive health risks which have grave consequences, including HIV/AIDS, STI, unwanted pregnancy and its complications. This study showed that the odd of having premarital sexual intercourse among students living out of campus were more likely compared to those living in the campus or dormitory. This might be due to high level of personal freedom and social interactions, which offers an opportunity for high level of sexual networking. This study revealed that those students who drink alcohol were more likely to practice premarital sexual intercourse compared to their counter parts. A study conducted in Nazareth is consistent with this finding [23]. This study reported that consumption of alcohol was high which is comparable with the findings of Maharaj et al who found out that 24% of adolescents in English-speaking Caribbean had used cigarettes [24] and John Imaledo et al who found that about 36% students of the University of Port Harcourt had history of current alcohol drinking [16]. This might be because adolescents’ substance abuse usually starts with alcohol and cigarette which are referred to as gateway substances. The easy accessibility of these substances to young people in most of our communities might be responsible for this high prevalence of its uses.

Those students who were stayed for two years in the campus were more likely to practice premarital sexual intercourse compared to first batch students. This finding is consistent with study conducted by Zubidia [25] and Sileshi [26] found that with an increase of educational level there is an increase in sexual practice. Communication on sexuality is very important. However, Parents think that communication on the subject of sexual issue can encourage children to be sexually active at earlier ages. This study found that those students Discussing sexuality and RH issue with family/relatives were more likely to practice premarital sexual than those not which is in contrary with A study conducted in Ethiopia by Adugna [27] found that 76.5% and 70.3% of the sexually active males and females were had very low communication with parents on sexual related issues. This may be due to the sensitivity of sexuality issues [28]. In this study, even if those students who had discussion on sexuality and RH were practice premarital sexual activity than those who were not, being discussing about sexuality and Reproductive health issue might increases their knowledge of reproductive Health risk reduction.

## Conclusion

In conclusion, the findings of this study showed the majority of the respondents were sexually active. Alcohol uses, Boarding and Discuss sexuality and RH issue with family/relatives were found significant predictors of premarital sexual practices. So there is the need to step up Reproductive health club at the university to bring behavior change among the students in order to detain the usual consequences of premarital sexual practices and risky sexual behavior.
